# Fit for purpose: perspectives on rapid reviews from end-user interviews

**DOI:** 10.1186/s13643-017-0425-7

**Published:** 2017-02-17

**Authors:** Lisa Hartling, Jeanne-Marie Guise, Susanne Hempel, Robin Featherstone, Matthew D. Mitchell, Makalapua L. Motu’apuaka, Karen A. Robinson, Karen Schoelles, Annette Totten, Evelyn Whitlock, Timothy J. Wilt, Johanna Anderson, Elise Berliner, Aysegul Gozu, Elisabeth Kato, Robin Paynter, Craig A. Umscheid

**Affiliations:** 1 Department of Pediatrics, University of Alberta, ECHA 4-472, 11405-87 Avenue, Edmonton, Alberta T6G 1C9 Canada; 2 Scientific Resource Center for the AHRQ Effective Health Care Program, Portland VA Research Foundation, 3710 SW U.S. Veterans Hospital Road, Portland, OR 97239 USA; 3 Evidence-based Practice Center, RAND Corporation, 1776 Main Street, Santa Monica, CA 90407 USA; 4 Alberta Research Center for Health Evidence, University of Alberta, Edmonton Clinic Health Academy 4-486D, 11405-87 Avenue, Edmonton, Alberta T6G 1C9 Canada; 5 ECRI-Penn AHRQ Evidence-based Practice Center, University of Pennsylvania Health System, 3535 Market St. Suite 50, Philadelphia, PA 19104 USA; 6 Department of Medicine, Johns Hopkins University, Baltimore, MD USA; 7 ECRI Institute, 5200 Butler Pike, Plymouth Meeting, PA 19462 USA; 8 Pacific Northwest Evidence-based Practice Center, 3181 SW Sam Jackson Park Rd, Portland, OR 97239 USA; 9 Kaiser Permanente Research Affiliates Evidence-based Practice Center, Center for Health Research, Kaiser Permanente Northwest, 3800N. Interstate Ave, Portland, OR 97227 USA; 10 Patient-Centered Outcomes Research Institute, 1919 M Street, Washington, DC 20036 USA; 11 Minnesota Evidence-based Practice Center, Minneapolis VA Center for Chronic Disease Outcomes Research and the University of Minnesota, 1 Veterans Drive, 111-0, Minneapolis, MN 55417 USA; 12 Agency for Healthcare Research and Quality, 5600 Fishers Lane, Rockville, MD 20857 USA; 13 Center for Evidence-based Practice, University of Pennsylvania, 3535 Market Street, Mezzanine, Suite 50, Philadelphia, PA 19104 USA

**Keywords:** Rapid reviews, Systematic reviews, Knowledge synthesis, Decision-makers, End-users, Interviews

## Abstract

**Background:**

There is increasing demand for rapid reviews and timely evidence synthesis. The goal of this project was to understand end-user perspectives on the utility and limitations of rapid products including evidence inventories, rapid responses, and rapid reviews.

**Methods:**

Interviews were conducted with key informants representing: guideline developers (*n* = 3), health care providers/health system organizations (*n* = 3), research funders (*n* = 1), and payers/health insurers (*n* = 1). We elicited perspectives on important characteristics of systematic reviews, acceptable methods to streamline reviews, and uses of rapid products. We analyzed content of the interview transcripts and identified themes and subthemes.

**Results:**

Key informants identified the following as critical features of evidence reviews: (1) originating from a reliable source (i.e., conducted by experienced reviewers from an established research organization), (2) addressing clinically relevant questions, and (3) trusted relationship between the user and producer. Key informants expressed strong preference for the following review methods and characteristics: use of evidence tables, quality rating of studies, assessments of total evidence quality/strength, and use of summary tables for results and conclusions. Most acceptable trade-offs to increase efficiencies were limiting the literature search (e.g., limiting search dates or language) and performing single screening of citations and full texts for relevance. Key informants perceived rapid products (particularly evidence inventories and rapid responses) as useful interim products to inform downstream investigation (e.g., whether to proceed with a full review or guideline, direction for future research). Most key informants indicated that evidence analysis/synthesis and quality/strength of evidence assessments were important for decision-making. They reported that rapid reviews in particular were useful for guideline development on narrow topics, policy decisions when a quick turn-around is needed, decision-making for practicing clinicians in nuanced clinical settings, and decisions about coverage by payers/health insurers. Rapid reviews may be more relevant within specific clinical settings or health systems; whereas, broad/national guidelines often need a traditional systematic review.

**Conclusions:**

Key informants interviewed in our study indicated that evidence inventories, rapid responses, and rapid reviews have utility in specific decisions and contexts. They indicated that the credibility of the review producer, relevance of key questions, and close working relationship between the end-user and producer are critical for any rapid product. Our findings are limited by the sample size which may have been too small to reach saturation for the themes described.

**Electronic supplementary material:**

The online version of this article (doi:10.1186/s13643-017-0425-7) contains supplementary material, which is available to authorized users.

## Background

Rapid reviews are a form of evidence synthesis that may provide more timely information for decision-making compared with standard systematic reviews. Systematic reviews are defined as “a review of a clearly formulated question(s) that uses systematic and explicit methods to identify, select, and critically appraise relevant research, and to collect and analyze data from the studies that are included in the review” [1]. While systematic reviews are comprehensive, they can take on average one to 2 years to complete [2, 3] and involve a substantial amount of resources to produce according to current standards [4]. Rapid reviews “are literature reviews that use methods to accelerate or streamline traditional [systematic review] processes” in order to meet the needs and timelines of the end-users (e.g., “government policymakers, health care institutions, health professionals, and patient associations”) [2].

In 2014, a project conducted through the Evidence-based Practice Center (EPC) Program of the U.S. Agency for Healthcare Research and Quality (AHRQ) explored the range of products that are considered rapid [5, 6]. AHRQ “is the lead federal agency charged with improving the safety and quality of America’s health care system” (http://www.ahrq.gov/cpi/about/profile/index.html). AHRQ produces evidence to improve the safety and quality of the U.S. health care system through its Effective Health Care Program (EHCP), and “works within the U.S. Department of Health and Human Services and with other partners to make sure that the evidence is understood and used” (http://www.ahrq.gov/cpi/about/profile/index.html). Institutions in the USA and Canada serve as EPCs under contract to produce evidence reports that are used to inform “coverage decisions, quality measures, educational materials and tools, clinical practice guidelines, and research agendas” (http://www.ahrq.gov/research/findings/evidence-based-reports/overview/index.html). The Scientific Resource Center (SRC) supports the EHCP by providing scientific and technical support to protect scientific credibility and independence of EHCP products (https://effectivehealthcare.ahrq.gov/index.cfm/who-is-involved-in-the-effective-health-care-program1/about-the-scientific-resource-center1/).

**Fig. 1:**
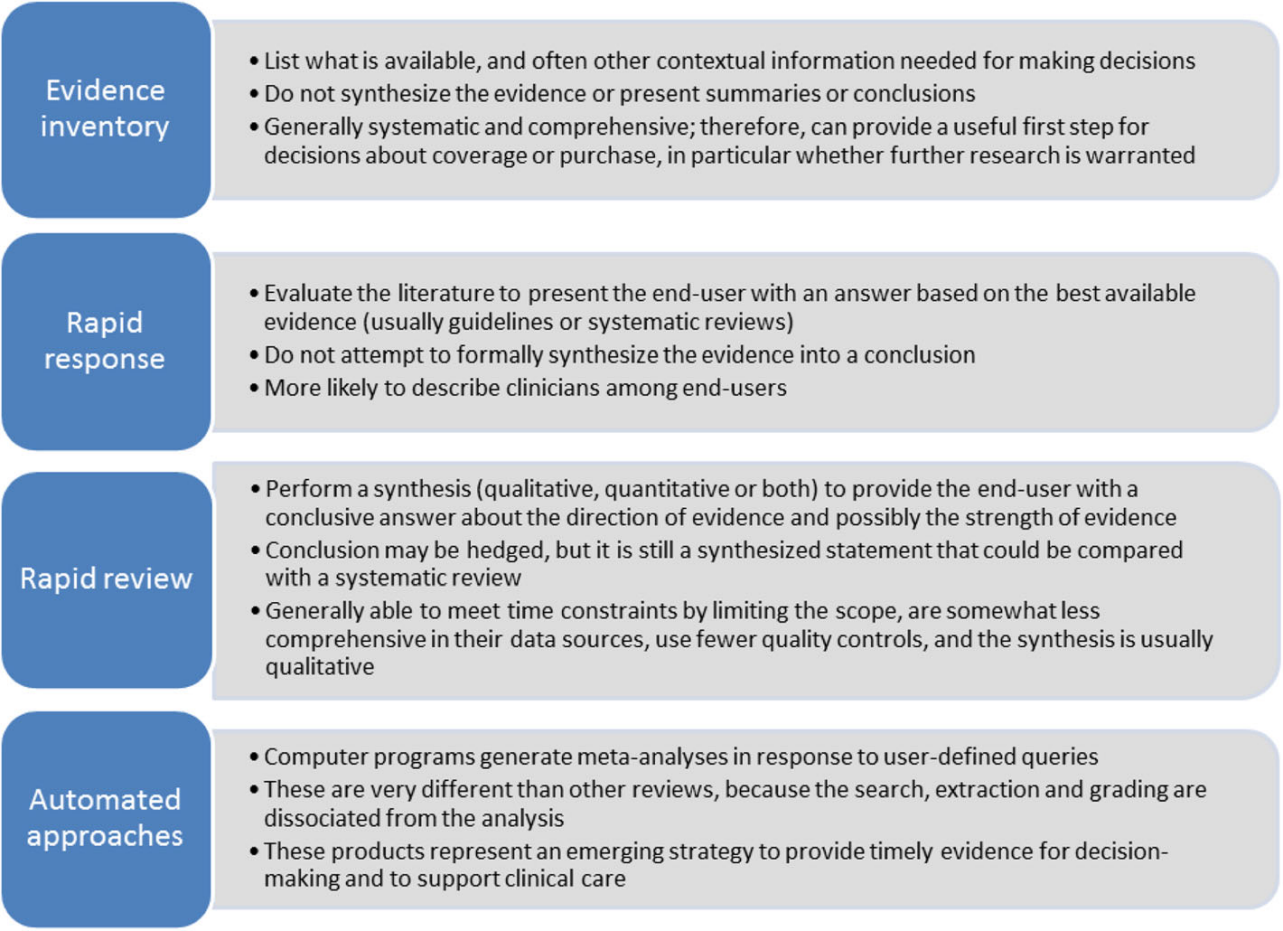
Taxonomy of rapid products. This taxonomy provides general descriptions of rapid products based on a previous analysis of 36 different rapid products from 20 organizations worldwide [5]. We recognize that there may be some overlap in individual products

In the 2014 project, we identified and examined 36 different rapid products from 20 organizations worldwide and interviewed 18 producers of rapid products [5]. A careful analysis led to a practical classification, or taxonomy, of rapid products according to the extent of synthesis (Fig. 1): inventories, rapid responses, rapid reviews, and automated approaches. A key finding was the observation, based on interviews with 18 producers of rapid reviews, that one of the biggest differences of rapid products compared with standard systematic reviews was the relationship between the review producer and the end-user. The paper noted that “rapid products are often conducted to help a specific end-user make a specific decision in an identified timeframe; therefore, the reviewers need to make decisions about what they can provide in the time allowed.” This often necessitates a direct relationship with a specific end-user with iterative feedback throughout the project to manage the scope (i.e., in terms of number and extent of research questions, outcomes, etc.) and agree on acceptable methodological approaches and trade-offs.

Our previous research focused on producers of rapid products; in the present project, we were interested in gaining an understanding of end-user perspectives on rapid products. We undertook the present work to investigate the acceptability to end-users of different rapid products and the context in which particular products may be useful. Our objectives were to determine:

1. What makes end-users trust and value an evidence synthesis, and whether this varies by the nature of the decision being made;
2. End-user impressions of different rapid products with a focus on acceptability and usability (e.g., impressions with respect to strengths and limitations; trade-offs in terms of approaches they would find acceptable to alter to increase efficiencies; and their perceptions of risks for answers being “wrong” or information that might be missed);
3. Where/when/how end-users might use rapid products and whether this varies by the nature of the decision being made.

## Methods

### General approach

A workgroup of 17 members from 8 EPCs, the SRC, and AHRQ participated in twice monthly teleconference calls over the course of 10 months to discuss project direction and scope, assign and coordinate tasks, collect and analyze data, and discuss and edit draft documents [7]. Workgroup members brought a range of clinical and methodological expertise and had extensive collective experience producing systematic reviews, other forms of evidence syntheses (including rapid products), and working with stakeholders to put evidence into practice. We undertook a qualitative study involving in-depth interviews to gather end-user perspectives of rapid products and identify themes. Qualitative interviews allow the interviewer to obtain the information sought, and due to their open-ended nature, they also provide the opportunity for the interviewer to probe for greater depth and clarity and for the respondent to elaborate or provide examples to illustrate concepts and perspectives [8]. Detailed methods and results are available in the full report [9].

### Key informant selection and interviews

To ensure that study findings would be relevant to the AHRQ EPC Program (the funder of this project) and that key informants would be familiar with standard review methods, we used purposive sampling and focused on frequent end-users of EPC reviews. Through discussion, the workgroup members identified different types of organizations that may use EPC reviews: research funders, payers/health insurers, health system/health care provider organizations, and societies/associations (e.g., that produce guidelines). We then identified 12 organizations representing these different stakeholders. We approached individuals from these organizations and invited them to participate in an interview (Additional file 1: Appendix A). Prior to the interviews, each key informant completed a conflict of interest form; no disclosed conflicts precluded participation. All key informants were familiar with or had used EPC reports (i.e., traditional systematic reviews). While we did not specifically select organizations or participants based on their experience with rapid products, some participants did have experience with rapid products.

One workgroup member (J-MG) conducted all interviews between January and March 2015 using a semi-structured interview guide designed to elicit a multi-faceted understanding of the value and uses of rapid products. The interviewer is a physician scientist with extensive experience in quantitative and qualitative methods, including the production of systematic reviews, stakeholder engagement, and in-person and telephone interviews. Prior to the interview call, participants were sent the interview questions and samples of rapid products (evidence inventory, rapid response, rapid review, and full EPC evidence report) that would be discussed. The interviews lasted approximately 1 hour and were attended by at least two additional workgroup members. All interviews were digitally recorded and transcribed verbatim.

### Interview guide

The workgroup developed the interview guide, consisting of open-ended questions (Additional file 1: Appendix B), through a review and discussion of multiple iterations. The workgroup discussed general concepts that we wanted to address in the interviews, several members of the workgroup drafted the interview guide, all workgroup members provided feedback, and we discussed with the full workgroup to ensure face validity. We wanted to understand how our key informants used traditional systematic reviews and what they valued in these products (e.g., what made the systematic reviews trustworthy or reliable, what components were most critical to informing their decisions) (objective 1). We were also interested in their impressions of the different rapid products (evidence inventory, rapid response, rapid review—see Fig. 1) (objective 2), as well as whether they would consider any of the rapid products useful and, if so, in what context (e.g., for specific decision-making needs) (objective 3). In particular, we wanted to know (a) what trade-offs they would be willing to accept to increase efficiencies in the production of reviews (objective 2), and (b) what risks they perceived might be incurred with rapid approaches (e.g., inaccurate findings, missing studies) (objective 2).

### Sample rapid products

From an initial list of 11 EPC systematic reviews addressing topics considered to be of general interest and completed in the last 2 to 3 years, we reached consensus and selected four potential review topics: venous thromboembolism, fecal DNA testing, pressure ulcers, and methicillin-resistant staphylococcus aureus. We then searched to identify rapid products on these topics through agencies we knew produce rapid products. We looked for examples that reflected the key categories of rapid products (inventories, rapid response, rapid review) identified in our previous work (Fig. 1) [5]. We found the highest number and the broadest range of rapid products for venous thromboembolism and chose among these to represent the three rapid products to share with the key informants. The evidence inventory was produced by the Canadian Agency for Drugs and Technologies in Health, [10] the rapid response was produced by ECRI Institute, [11] and the rapid review was produced by the Penn Medicine Center for Evidence-based Practice [12, 13]. These three organizations have extensive experience producing rapid products. The full EPC systematic review on the same topic is available on the AHRQ website [14].

### Data analysis

Transcripts were independently analyzed using content analysis with NVivo^TM^ software by two investigators trained in qualitative analyses (MLM, JA). Two other investigators with experience in qualitative analysis independently reviewed all transcripts and verified themes and subthemes (J-MG, LH). To ensure reliability of the coding structure, all themes and subthemes were also reviewed in the larger multi-disciplinary workgroup.

We adhered to methodologically sound qualitative research methods including independent reviews of texts, thematic coding by qualitatively-trained reviewers, and convergence of interpretation across a multi-disciplinary team of researchers. Our analysis was guided by principles of grounded theory, wherein an understanding of the concept of interest arises from the empirical data rather than from a priori hypotheses. Two trained qualitative analysts independently analyzed responses to identify and code themes and subthemes. After a preliminary review of the data, the investigators developed an initial list of codes, which were modified and expanded as the analysis progressed. To ensure reliability of the coding structure, all themes and subthemes were reviewed with the larger multi-disciplinary research team which included experts in evidence reviews, rapid reviews, stakeholder engagement, clinical care, and research.

## Results

A total of eight interviews were conducted with U.S. organizations representing: guideline developers (*n* = 3), health care providers (*n* = 3), research funder (*n* = 1), and non-commercial payer/insurer (*n* = 1). Four individuals who were invited to participate did not respond. For one of the organizations represented by those who did not respond, we approached two alternate potential participants who declined as they felt their input would not be relevant or helpful. Finally, one individual who had agreed to participate had to withdraw because of a last-minute scheduling conflict.

Six of eight key informants had clinical backgrounds; of these, two produced guidelines for professional organizations, two represented health systems, one was a funder, and one represented a non-commercial payer. Two key informants were non-clinical; one produced guidelines for a professional group and the other represented a health system. All key informants routinely commissioned and used systematic reviews. There was varied representation with respect to knowledge and use of rapid review products: three key informants were involved in producing rapid review products, two key informants used rapid review products, and three key informants were unaware of or had no experience using rapid review products.

### Users’ perspectives on important characteristics of reviews (objective 1)

Key informants were asked what they considered the most important characteristics of review products for their use. Themes that emerged (Table 1) were related to methods, source of the review, relationship between producer and user, clinical significance of the review questions, and the recency of the review. The most commonly reported review characteristics deemed critical were (1) the review was conducted by a reliable source, i.e., conducted by experienced reviewers from an established research organization (all key informants); (2) the review addressed clinically relevant questions (all key informants); and (3) there was a relationship between the user and producer of the review (seven of the eight key informants; one key informant did not discuss). Other important review characteristics that were noted included recency of the review and use of sound methodology (each item was noted by four key informants and not mentioned by four key informants).

**Table 1:** Key informant interviews: important characteristics of reviews

Element	Theme	Sample quotes [type of end-user]
Methods	Important that sound methods are used in developing review	“..adherence to good standards of evidence evaluation is really critical, so that probably matters more to me than anything” [provider]
Source	Trust review products from reliable sources	“If it came from a place that we trust then we would have more confidence in using it than if it was just arbitrarily out there from somebody who had done it once.” [guideline developer] “the source is always really important, knowing that someone is evaluating the evidence in a rigorous way, the way that we do and the way the evidence based practice centers do means a lot” [provider]
Relationship between producer/user	Important to establish relationship with user up front	“… the quality that we've had in the reviews when they have that connection up front is significantly different…I think it also helps build trust in how the evidence is being done.” [guideline developer] “…I think it’s incredibly important for the guideline developers to be involved from day one.” [guideline developer]
Clinical significance	Reviews should include considerations of clinical importance, not just statistical significance	“…ultimately, the clinical aspect is important. There is sometimes a gap between the statistical significance versus what’s clinically significant…” [guideline developer]
Recency	Important that a report is recent. A gap search is sometimes done	‘We’re usually hoping we find something within the last two to three years” [guideline developer] “We’re certainly willing to do the bridge look search to make sure that there hasn’t been something big that’s come up that might adjust the estimated treatment effects” [guideline developer]
Key questions	The framing of the question can be the most important aspect of a review It is important that the key questions address what the end-user needs, including clinical outcomes and consideration of benefits and harms Narrowing the scope of the key questions can sometimes be problematic	“…the thing that I find most helpful in this approach to evidence always is the framing of the question” [provider] “…we want to make sure that the questions that were addressed are what the guideline developers interested in terms of just plain old clinical outcomes” [guideline developer] “When I’ve used some other Rapid Reviews, when they narrow the scope they probably at least half the time completely miss the mark of the question we want answered. Keeping it a little bit broader would be something that I would not sacrifice…” [payer]

### Users’ perspectives on review methods (objective 2)

Table 2 presents themes and sample quotes regarding the key informants’ perspectives on individual components of a review. Key informants felt that the following were very important: quality rating of studies (noted by seven key informants; not mentioned by one key informant), data tables (including characteristics of included studies) (noted by four key informants; not mentioned by four key informants), assessment of overall quality/strength of evidence (noted by five key informants; not mentioned by three key informants), and use of summary tables of results and conclusions (noted by three key informants; not mentioned by five key informants). One key informant (research funder) mentioned that specific recommendations regarding future research needs were important for use in research development and funding.

**Table 2:** Key informant interviews: perspectives on review methods

Element	Theme	Sample quotes [type of end-user]
Literature search	*It is okay to limit the search by database, journal, year, etc.* as long as it is scientifically justified	“I’d probably be more comfortable with selecting top 20 [journals]…and just do the evidence review using those.” [guideline developer] “I would not expect things like looking for unpublished literature.” [payer] “you…probably you get 90% or 95% of the evidence with 20% to 30% of the searching” [provider]
Abstract/full-text screening	*It is acceptable to have single review of abstracts and full text*	“To me that [single review] would be acceptable.” [research funder] “I think implicitly in these kinds of rapid reviews…you’re going to do a combination of looking at existing reviews so that will help catch stuff that you might otherwise miss with single review.” [provider]
Quality assessment	Some assessment of literature quality is necessary	“I think that [quality assessment] should be included.” [payer] “it’s important that we probably would want some level of comment on that [quality assessment]” [provider]
Data tables/extraction	Evidence tables are useful	“I think the most important part of an evidence review is always going to be the evidence tables” [guideline developer] “one part that we use a lot are of course the extraction tables” [guideline developer]
Quality/strength of evidence	Quality/strength of evidence is important	“That [strength of evidence grading] would be very important.” [payer] “The strength of the evidence I always find valuable as well.” [provider]
Summary tables	Summary tables or ways to present the results/conclusions in an accessible format are useful	“A lot of times you’ll do good summary tables, and that’s probably where I would look…” [payer] “The work development teams in our clinical programs are more concerned with what’s the summary of the evidence.” [provider]
Future research recommendations	Future research recommendations are helpful for research development	“…what are the future research recommendations…99.9% of the systematic reviews all concluded more research is needed, so focus on exactly what are they recommending.” [research funder]

Comments that are italicized represent most acceptable trade-offs

The most commonly reported acceptable trade-offs to increase efficiencies were associated with the literature search (all six key informants who mentioned literature search agreed that limits on such items as date or language were acceptable) and citation and full-text review (among five key informants who discussed this point, four agreed that a single review was acceptable, and one agreed depending on the expertise of the individual doing the review). The majority of key informants were willing to accept shortcuts made in these areas in exchange for a shorter production time.

### Users’ perspectives on uses of rapid products (objective 3)

Key informants reported a variety of potential uses for rapid products and standard systematic reviews (Table 3). Several stated that the evidence inventory (three key informants; guideline developers, provider organization) and rapid response (four key informants; guideline developers, research funder, provider organization) would not be useful for their purposes, while other key informants considered these useful for new topics, to understand the depth and/or breadth of existing evidence/available literature, for restricted local use, or for clinicians who are already familiar with the literature in a topic area. When key informants did see utility in the rapid products, they were more often viewed as interim products (or “placeholders”) to inform downstream investigation (e.g., whether to move forward with a full review or a guideline, direction for future research/funding); the rapid products were viewed less commonly as useful for “end-point” decisions (especially the evidence inventory and rapid response). Moreover, some key informants commented that the level of detail available in the evidence inventory and rapid response was not sufficient for decision-making; most key informants indicated that analysis/synthesis and assessment of overall quality/strength of evidence was important to this end.

**Table 3:** Key informant interviews: uses of rapid products and standard systematic reviews

Use	Evidence inventory	Rapid response	Rapid review	Systematic review
For broad topic areas/population issues				X
To inform research agenda				X
For in-depth understanding of a topic area				X
For guideline or recommendation development			X	X
For guideline/recommendation updates or new issues subsequent to a guideline/recommendation			X	X
For coverage decisions			X	X
For organizational or policy change			X	X
For implementation			X	X
For quick decisions			X	
When no previous SR or guidance exists			X	
For “hot” or timely topics	X	X		
In area with limited literature	X	X		
To understand depth and/or breadth of evidence e.g., evidence maps	X	X		
To clarify whether a review is already available	X	X		
To ignite/catalyze change or challenge the status quo	X	X		

All but one key informant indicated that they could see a use for a rapid review, in particular, for guideline development (particularly for narrow topics), policy decisions when a quick turn-around is needed, decision-making in nuanced clinical settings and for practicing clinicians, and coverage decisions. Most key informants felt that standard systematic reviews cannot be used for quick decisions, unless they already exist, in which case they often need a bridge search. Key informants noted factors to be considered when using a rapid review, including that the review must (a) have quality methods and/or be from a reliable source and must also (b) address specific questions/clinical outcomes of interest. Generally, a traditional systematic review is preferred but in cases where none exists, Key informants are willing to accept rapid reviews for shorter turn-around. Key informants noted limitations with rapid reviews in terms of evaluating the spectrum of benefits and harms often needed for their decision-making purposes, and limited detail with respect to important subgroups.

Another theme that emerged is that rapid responses and rapid reviews may be more relevant for issues (often narrow questions) that arise within the clinical setting specific to a health system (where it may be more feasible to narrow the scope), or when interest is focused more on implementation (e.g., tailoring the evidence to a given region/setting). Conversely, key informants felt that broad/national guidelines more often need a full systematic review. The following comment highlights this perspective (guideline developer):

So that’s the dichotomy to me is that there are people working on implementation and what’s going to happen out in our system of care delivery; and there are people working on trying to make sure that what we’re saying is the right thing to be doing and there’s a tension between that. We’ve often talked about the struggle to balance rigor with efficiency and so the operationally-oriented folks are willing to take a risk on the absolute correctness and the answer in order to do something and to do it reliably across the delivery system where the more traditional EBM people and guideline people would really rather take the time to make sure they know that they’ve got the right answer.

Many of the key informants emphasized that an evidence review is just one part of the decision-making process. There are many other factors considered, including cost and feasibility.

## Discussion

Our study provides information about how end-users perceive different types of rapid products and what features are the most important (Table 4). We specifically asked key informants who use AHRQ-sponsored systematic reviews about three different rapid review types (evidence inventory, rapid response, and rapid review; Fig. 1). Our results suggest that each product type could prove useful under specific circumstances; further, key informants were more likely to consider them useful if they had previous direct experience using them. The following observations were made for the specific rapid review products.

- *Evidence inventory*: Although some of the key informants saw value in this product, it was generally not considered sufficient to inform decision-making because it did not “give an answer to the question” or a synopsis of the evidence. Some key informants indicated that an evidence inventory may be useful to stimulate discussion, to challenge the status quo, or to get a sense of the literature when there is a pressing concern. These situations were typically in the context of a hospital system.
- *Rapid response*: Few of our key informants found this product to be sufficient for their decision-making needs although they did prefer this to an evidence inventory. A perceived use for a rapid response was to validate the need for future research or a rapid or traditional review (e.g., identify the volume of research in a given area and whether there is consistency in terms of benefits).
- *Rapid review*: Most of the key informants liked rapid reviews and generally considered this to be acceptable when a traditional systematic review is unavailable. While guideline developers generally wanted something more comprehensive and detailed, they felt rapid reviews may be used for guidelines on narrow topics, updates of guidelines, or for new issues that arise subsequent to a guideline or recommendation. Other end-users (e.g., payer/insurer) were less concerned about comprehensiveness. Positive aspects of the rapid review included its conciseness and a focus on existing syntheses and high quality studies. This was considered useful for policy decisions that were time-dependent.

**Table 4:** Key informant interviews: themes about review products

Category (features of the producer, report, or decision)	Theme	Description
Producer	Trust	• This was the primary issue that arose in the context of how end-users valued a review, and whether they would rely on a rapid product • Methodological alterations appear to be secondary to the trust established through consistent products and active end-user engagement
Producer	Close working relationship	• Maintaining a close working relationship between the end-user and the review producer was considered important to ensure that the key questions reflected the end-user’s needs
Report	Relevance of the key questions	• Key informants stressed this, noting that if questions did not directly address the specific end-users’ needs, the review was of little or no value, regardless of the methods used
Report	Quality/strength of evidence and evidence tables	• Several key informants found these elements to be essential and often the most valuable part of the reports • End-users liked to see outcome data, individual study quality, and overall quality/strength of evidence assessment summarized in a readily accessible form
Report	Responsibility of reviewers to highlight methodological considerations/limitations	• Reviewers need to help users understand potential ramifications of streamlined methods as end-users may not be aware of standard review steps and accepted methodological approaches
Decision	Ability to easily change or reverse a decision	• May be one hallmark of when a rapid product is useful • For example, key informants expressed that a full systematic review is more often necessary for clinical practice guidelines, broad application of the evidence (e.g., “change the direction of the organization on a very important topic”), or macro topics (e.g., population-level implementation) • Conversely, a rapid product may be sufficient: for decisions being made on a local basis (e.g., point-of-care clinical decisions, nuanced clinical situations, local coverage decisions) where there is not the same level of scrutiny; for “in the moment sort of decisions”; to act as an update for a previous comprehensive guideline or address an issue that comes up secondary or subsequent to a guideline; or to get a general sense of the literature or scale of the issue
Decision	There is generally more than the evidence of benefits and harms to consider when making a decision	• Rapid products provide one source of information among an array of other considerations for decision-making • Other factors include context and varied viewpoints, the burden of disease and population affected, and costs. • Due to these other factors, there may be less perceived risk of using a rapid product

Our findings reaffirm that an interactive and ongoing relationship ensures that products meet end-user’s needs, particularly for the development of key questions that the review aims to answer [15]. Further, our findings confirm that trust is an important factor in the usability of rapid reviews, and this trust is based on both the established reputation of the producer and on how the producer works with the end-user iteratively over the course of refining/scoping the topics and producing the review or reviews to meet their specific decision-making needs. This places the responsibility for the reliability and validity of the product in the hands of the producers. Producers need to ensure that rapid products use transparent methods that communicate potential limitations and risks to end-users [16]. In addition, producers should be aware of the potential harm that a misleading conclusion from a rapid product might have on their reputation [17].

End-users are willing to accept trade-offs in review methods, such as limiting the literature search and conducting single screening of abstracts and full text, in order to get more timely information to support decisions. For the scientific systematic review community, eliminating key procedures meant to reduce reviewer errors and bias represents an important variation from standard SR methodology [18]. This raises two important questions that can only be answered through empirical research. First, to what extent do these shortcuts reduce the validity of the final conclusion? There has been limited empirical research on the incremental validity of systematic review methods [19–24]; moreover, very few studies have compared the conclusions reached in rapid reviews vs. systematic reviews [25, 26]. A second empirical question is how much time is actually saved by different methodological shortcuts. For instance, our key informants agreed that limits to the literature search would be acceptable (e.g., date or language restrictions); however, it is not known how much time this would actually save. We recognize the challenge of conducting empirical comparisons of SRs and rapid products, particularly given the variability in topics and contexts in which both are generated. If it is feasible to collect these kinds of data, it may be possible to make more informed decisions about the benefits and risks of rapid review products in different contexts.

According to our key informants, other characteristics of the review product also appear to significantly contribute to the usability of the product, such as quality assessment of included studies (payer and provider), the use of evidence and summary tables (guideline developers), assessment of the quality/strength of the evidence base as a whole (payer and provider), and future research recommendations (research funder). The trade-off of reducing or removing these aspects of reviews to gain efficiencies may result in the review being less valued by some end-users. Further, if all these elements are desired (or required), it may be more likely that reviewers can gain efficiencies through focusing the scope and questions of the review.

### Strengths and limitations

We followed sound qualitative methodology in eliciting perspectives from different types of users and in identifying themes. However, our small sample size (ideally 12–15 in qualitative research to identify patterns [10]) means that results are likely not representative of all end-users and we cannot be sure that the themes reached saturation. This is especially true in considering whether there are differences in the perspectives across and between types of users. Further, key informants included some individuals who had not used rapid products; therefore, their perspectives on the value and uses of rapid products are hypothetical. The hypotheses generated from this work need further evaluation. A further limitation is our selection of a sample of rapid products from known producers. Our sample of products was chosen based on our knowledge of the various rapid products and methods gained through our previous research [5]. Informants’ views may not be generalizable to all rapid products or those on other topics.

We chose to interview frequent and known users of AHRQ comprehensive systematic evidence review products. It may be that infrequent users or more varied audiences would have different perspectives. For instance, users with less experience in traditional systematic review methodology would perhaps find fewer limitations and more value in rapid review products. Eliciting views from additional audiences, such as those for whom the rapid products were designed, may also help in understanding obstacles or benefits to using rapid review products. However, current users of AHRQ products provide a critical perspective as they are knowledgeable about systematic review methodology, have a high standard for evidence synthesis, and may be considered a key audience for rapid review products.

### Future directions

We did not systematically compare the needs and values of different end-users; further, there are other perspectives we did not collect, including those from frontline clinicians, patients, and infrequent users of AHRQ products. Conducting interviews or focus groups with a larger number of more diverse users and incorporating patient perspectives would allow differences to be explored and enable the evaluation of hypotheses identified in this work. Interviewing a range of stakeholders at the same time, and having informants share their different decision-making contexts, may elicit important contrasts and insights about the usefulness of rapid products.

We were also unable to identify specific trade-offs that would be acceptable to end-users. A more structured survey of end-users, as well as producers, may provide more information about trade-offs. These types of questions could elucidate what would be acceptable in terms of time or other resource trade-offs for the inclusion of a specific review methodology or characteristic. Under what circumstances, for what questions, would end-users trust a specific type of rapid review product? The key to collecting valid information is that end-users understand the steps involved in a review as well as empiric evidence of the impact of alternative methods on results and conclusions.

Ideally, future studies would also move beyond hypothesis generation towards empirical testing. One possible design is the completion of systematic reviews and rapid review products on the same questions; a study of this nature, the SPARKS Study, has recently been funded by the Canadian Institutes of Health Research (http://webapps.cihrirsc.gc.ca/cris/detail_e?pResearchId=7016266&p_version=CRIS&p_language=E&p_session_id=2514166). Ultimately, we would want to know how long it takes to produce each product, whether these different review products were more or less useful for end-users in informing decisions, and most importantly, if those decisions would be different depending on the product used.

Empirical research on the impact of streamlining specific methodological approaches is essential given the finding that changes to methodological approaches appear to be acceptable to end-users as long as trust is established through consistent products and active end-user engagement, and there is clear communication by producers about methods and the potential ramifications of streamlined methods.

Finally, this and our previous work on rapid reviews have identified a wealth of information contained within these rapid products that could be useful to stakeholders beyond those commissioning the specific rapid reports. However, there is no central repository for rapid products. Many are not published in traditional peer-reviewed sources, indexed in bibliographic databases, or digitally archived. While some are publically available on the websites of review producers, others are only available upon request or subscription. Discussion among the community of rapid review producers to explore the potential for a central repository is warranted [27].

## Conclusions

Evidence inventories, rapid responses, and rapid reviews have utility in specific decisions and contexts. For any rapid product, the credibility of the review producer, relevance of key questions, and close working relationship between the end-user and producer are critical.

## Additional file

Additional file 1: Appendix A (Invitation to key informants) and Appendix B (Interview guide). (DOCX 28 kb)

## Abbreviations

AHRQ: Agency for healthcare research and quality
EPC: Evidence-based practice center
KI: Key informants
SR: Systematic review

## References

[1] Higgins JP, Green S (editors). Cochrane handbook for systematic reviews of interventions Version 5.1. 0 [updated March 2011]. Cochrane Collaboration. 2011. Available from: https://www.handbook.cochrane.org.

[2] Ganann R, Ciliska D, Thomas H (2010). Expediting systematic reviews: methods and implications of rapid reviews. Implement Sci.

[3] Sampson M, Shojania KG, Garritty C, Horsley T, Ocampo M, Moher D (2008). Systematic reviews can be produced and published faster. J Clin Epidemiol.

[4] Chandler J, Churchill R, Higgins JP, Lasserson T, Tovey D. Methodological standards for the conduct of near Cochrane Intervention Reviews: Cochrane Collaboration2013 December Contract No.: Version 2.3.

[5] Hartling L, Guise J, Kato E, Anderson J, Aronson N, Belinson S et al. EPC Methods: An Exploration of Methods and Context for the Production of Rapid Reviews. Research White Paper..(Prepared by the Scientific Resource Center under Contract No 290-2012-00004-C) AHRQ Publication No 15-EHC008-EF Rockville, MD: Agency for Healthcare Research and Quality; February 2015: www.effectivehealthcare.ahrq.gov/reports/final.cfm. 25654160

[6] Hartling L, Guise J-M, Kato E, Anderson J, Belinson S, Berliner E (2015). A taxonomy of rapid reviews links report types and methods to specific decision-making contexts. J Clin Epidemiol.

[7] Guise J-M, Chang C, Viswanathan M, Glick S, Treadwell J, Umscheid CA (2014). Systematic reviews of complex multicomponent health care interventions.

[8] Morse JM, Field PA (1995). Qualitative research methods for health professionals.

[9] Hartling L, Guise J, Hempel S, Featherstone R, Mitchell MD, Motu’apuaka ML et al. EPC Methods: AHRQ End User Perspectives of Rapid Reviews. AHRQ Publication No16-EHC014-EF Rockville, MD: Agency for Healthcare Research and Quality; April 2016: www.effectivehealthcare.ahrq.gov/reports/final.cfm. 27195347

[10] Canadian Agency for Drugs in Technologies in Health. Acetylsalicylic acid for venous thromboembolism prophylaxis: an update of clinical evidence. CADTH; 2014. Available from: https://www.cadth.ca/acetylsalicylic-acid-venous-thromboembolism-prophylaxis-update-clinical-evidence.

[11] ECRI Institute. Knee-length versus Thigh-length Compression Devices for Deep Venous Thrombosis. Plymouth Meeting, PA: ECRI Institute; 2012.

[12] Mitchell MD, Umscheid CA. Intermittent pneumatic compression devices for venous thromboembolism prophylaxis. Philadelphia: University of Pennsylvania Health System Center for Evidence-based Practice; 2009.

[13] Jayakumar KL, Lavenberg JA, Mitchell MD, Doshi JA, Leas B, Goldmann DR et al. Evidence synthesis activities of a hospital evidence‐based practice center and impact on hospital decision making. J Hosp Med. 2016;11(3):185–92. 10.1002/jhm.2498 26505618

[14] Sobieraj DM, Coleman CI, Tongbram V, Lee S, Colby J, Chen WT et al. Venous thromboembolism prophylaxis in orthopedic surgery. Comparative Effectiveness Review No. 49 (Prepared by the University of Connecticut/Hartford Hospital Evidence-based Practice Center under Contract No. 290-2007-10067-I.) AHRQ Publication No. 12-EHC020-EF. Rockville, MD: Agency for Healthcare Research and Quality March 2012: www.effectivehealthcare.ahrq.gov/reports/final.cfm.

[15] Danz MS, Hempel S, Lim Y-W, Shanman R, Motala A, Stockdale S et al. Incorporating evidence review into quality improvement: meeting the needs of innovators. BMJ Qual Saf. 2013;22(11):931–39. 10.1136/bmjqs-2012-001722 PMC3812883 23832925

[16] Featherstone RM, Dryden DM, Foisy M, Guise J-M, Mitchell MD, Paynter RA (2015). Advancing knowledge of rapid reviews: an analysis of results, conclusions and recommendations from published review articles examining rapid reviews. System Rev.

[17] Oxman AD, Schünemann HJ, Fretheim A (2006). Improving the use of research evidence in guideline development: 8. Synthesis and presentation of evidence. Health Res Policy Syst.

[18] Shea BJ, Hamel C, Wells GA, Bouter LM, Kristjansson E, Grimshaw J (2009). AMSTAR is a reliable and valid measurement tool to assess the methodological quality of systematic reviews. J Clin Epidemiol.

[19] Dickersin K, Scherer R, Lefebvre C (1994). Identifying relevant studies for systematic reviews. BMJ.

[20] Giustini D, Boulos MNK (2013). Google Scholar is not enough to be used alone for systematic reviews. Online J Public Health Inform.

[21] Moher D, Klassen TP, Schulz KF, Berlin JA, Jadad AR, Liberati A (2000). What contributions do languages other than English make on the results of meta-analyses?. J Clin Epidemiol.

[22] Selph SS, Ginsburg AD, Chou R (2014). Impact of contacting study authors to obtain additional data for systematic reviews: diagnostic accuracy studies for hepatic fibrosis. System Rev.

[23] Bushman BJ, Wells GL (2001). Narrative impressions of literature: the availability bias and the corrective properties of meta-analytic approaches. Personal Soc Psychol Bull.

[24] Horsley T, Dingwall O, Sampson M. Checking reference lists to find additional studies for systematic reviews. Cochrane Database Syst Rev. 2011, Issue 8 Art No: MR000026. doi: 10.1002/14651858.MR000026.pub2. 2011. 10.1002/14651858.MR000026.pub2 PMC7388740 21833989

[25] Watt A, Cameron A, Sturm L, Lathlean T, Babidge W, Blamey S (2008). Rapid reviews versus full systematic reviews: an inventory of current methods and practice in health technology assessment. Int J Technol Assess Health Care.

[26] Watt A, Cameron A, Sturm L, Lathlean T, Babidge W, Blamey S (2008). Rapid versus full systematic reviews: validity in clinical practice?. ANZ J Surg.

[27] Booth A, Wright K, Outhwaite H (2010). Centre for Reviews and Dissemination databases: value, content, and developments. Int J Technol Assess Health Care.

