# Supplementary material for: Fit for purpose: perspectives on rapid reviews from end-user interviews
Source: Syst Rev. 2017 Feb 17;6:32. doi: 10.1186/s13643-017-0425-7 (PMC5316162; doi:10.1186/s13643-017-0425-7)
Supplement: Additional file 1: — Appendix A (Invitation to key informants) and Appendix B (Interview guide). (DOCX 28 kb) [file 13643_2017_425_MOESM1_ESM.docx]

**Appendix A. Invitation to Key Informants**

Dear [Key Informant],

We are conducting a project on end-user perspectives of rapid reviews. This is an extension of a white paper we produced last year that examined methods and guidance for conducting rapid reviews. As part of this Agency for Healthcare Research and Quality (AHRQ) funded project, we are having discussions with thought leaders in the field who use AHRQ Evidence-based Practice Center (EPC) reviews.

Because of your experience as an end-user of EPC reviews, we would like to schedule a time to speak with you. If you or your organization does not use EPC reviews, please let us know. Also, please let us know if there is a different person in your organization that you think we should contact instead.

Your participation would involve a 60-minute individual interview. In this discussion we hope to learn your perceptions of evidence syntheses currently produced through the EPC Program, what aspects of them are helpful for your decision-making, and whether there are trade-offs in methods or comprehensiveness you are willing to make for different decisions and to meet different timelines.

**If you are able to participate, please respond to our doodle poll with your availability at [link to doodle poll]**

If you are unable to make any of the above times, please let us know and we may be able to arrange another meeting time.

**Please confirm whether or not you will be able to participate in this project by [Date, 2014].**

Thank you for your consideration. If you have any questions, or would like additional information, please contact Pua Motu’apuaka at [Makalapua.Motu’apuaka@va.gov](mailto:Johanna.Anderson2@va.gov) or 503.220.8262 x52367.

Sincerely,

Jeanne-Marie Guise, M.D., M.P.H.

Associate Director
Scientific Resource Center for the
AHRQ Effective Health Care Program

*Sent on behalf of Jeanne-Marie Guise by the AHRQ Scientific Resource Center*

Appendix B. Interview Guide

**Introduction**

The overall mission of the Agency for Healthcare Research and Quality’s (AHRQ) Effective Health Care (EHC) Program is to provide evidence-based information to health care stakeholders that is relevant to their needs, timely, objective, scientifically rigorous, and developed and presented with transparency.

**Objectives**

1. Determine what makes AHRQ end-users trust and value an evidence synthesis, including (but not limited to) extent of synthesis, extent of information, specific pieces of information, formatting/presentation of information, organization that produced the report (and their relationship with that organization), methods used to conduct the synthesis; does this vary by the nature of the decision being made?
2. Determine end-user impressions of different rapid products with a focus on acceptability and usability (not necessarily validity). Determine their impressions with respect to: strengths and limitations, trade-offs (what pieces or methods could be altered to increase efficiencies, what are they willing to accept), risks (in terms of the answer being ‘wrong’; how bothered that some information might be missed), where/when/how they might use them; does this vary by the nature of the decision being made; some guiding questions:
   1. If you had a rapid product, how would it be useful to you and in what context?
   2. If you had a time dependent decision, what are you confident using, knowing that different products may give different answers?

There are no right or wrong answers, so please feel free to share your thoughts openly. We would welcome any materials that you would like to share with us either before or after the discussion session. Please send any questions or materials to [Makalapua.Motu’apuaka@va.gov](mailto:makalapua.motu'apuaka@va.gov)

**Ground rules for discussion session**

The discussions will be tape recorded, transcribed, and analyzed for overarching themes. Although the report may list individuals who were interviewed, answers will not be identifiable to individuals or specific organizations. You may refrain from answering any questions and are welcome to leave the discussion at any time.

**Materials provided for discussion during the call**

We have sent you an example of a report produced through the Evidence-based Practice Center (EPC) program. We have also provided samples of rapid review products. During our discussion we will refer to these products. The following are the documents sent to you (these are further described in the Table on the last page of this interview guide):

- AHRQ EPC Comparative Effectiveness Review.pdf
- Evidence Inventory Sample.pdf
- Rapid Response Sample.pdf
- Rapid Review Sample.pdf

**Questions**

1. We have provided you with an example of a typical EPC report. Can you tell us how you have used or might use such a report?
2. In what context have you used or would you use a report like this (for what types of decisions, etc.)?
3. What elements of the report do you consider important/critical to informing decisions? E.g., type/breadth of questions; extent of search (number of databases, grey literature, date, setting, language); all outcomes versus select outcomes; quantitative results, forest plots; summary of findings / strength of evidence / GRADE tables; appendices (study details); how much do the details of individual studies matter, e.g., quality of primary studies; conclusions (do you find the conclusions helpful or prefer to draw your own); other
4. Under what circumstances have you or would you:
   1. Retrieve any of the individual studies
   2. Complete additional analyses
   3. Complete additional syntheses
5. Do you have knowledge of or experience using rapid review products?

*If you have experience using rapid review products:*

- 1. What kind of decision(s) did you make?
  2. What elements of the report were important/critical to informing your decision?
  3. Did you conduct additional analyses or gather additional information?
  4. Were there major benefits or limitations with the information available?
  5. Did you share with colleagues (where and who within the organization, e.g., individual physicians, committees, etc.)?
  6. What do you consider the pros and cons of using rapid review products?

*If you have knowledge of (but no experience using) rapid review products:*

- 1. What types of decisions do you think they would be helpful for?
  2. In general what do you consider the pros and cons of rapid review products?

1. Can you take a look at the sample of rapid review products we provided (see Table and attachments):
   1. Would you find any of these useful? If so, for what types of decisions?
   2. If you had a time-dependent decision, what type of product would you be willing to accept?
      1. If you requested a customized report, what would you be willing to trade-off to get your report in a timely fashion?
      2. If you were able to access a rapid product that someone else had commissioned, how useful would it be to you?
   3. Would you trust the information? Why or why not? What would increase your trust/confidence in the information?
   4. What pieces of information did you look for to tell us whether you would use it and/or trust it?
   5. What do you see as potential risks of using different types of synthesis products, or information generated using variable methods?
      1. If you need to make compromises in terms of the comprehensiveness and formatting of evidence, does the level of risk/compromise you are willing to make change based on the type of decision?
      2. What are factors you would consider in the risk you are willing to take (e.g., safety concerns, burden of disease, cost)?
      3. If the evidence is wrong, what is the acceptable level of risk (e.g., permanent vs. transient adverse effects)?
2. How important is the relationship with the producer of the evidence synthesis product?
   1. In terms of providing useful information to make your decisions
   2. In terms of credibility

Examples of Different Types of Rapid Review Products (See attachments sent in advance of the call)

| **Rapid Review Product** | **Document name** | **Title** |
| --- | --- | --- |
| **Evidence inventories** list what evidence is available, and often other contextual information needed for making decisions, but do no synthesis and do not attempt to present summaries or conclusions. | Evidence Inventory Sample | Acetylsalicylic Acid for Venous Thromboembolism Prophylaxis: an Update of Clinical Evidence |
| **Rapid responses** organize and evaluate the literature to present the end-user with an answer based on the best available evidence but do not attempt to formally synthesize the evidence into a new conclusion. Usually this means reporting the conclusions of guidelines or systematic reviews, but some rapid response products apply a best evidence approach and report the results of primary studies if no secondary sources are available. | Rapid Response Sample | Knee-length versus Thigh-length Compression Devices for Treating Deep Venous Thrombosis |
| **“True” rapid reviews** perform a synthesis (qualitative, quantitative, or both) to provide the end-user with an answer about the direction of evidence and possibly the strength of the evidence. | Rapid Review Sample | Intermittent Pneumatic Compression Devices for Venous Thromboembolism Prophylaxis |
